# Effect of human activated NRAS on replication of delNS1 H5N1 influenza virus in MDCK cells

**DOI:** 10.1186/1743-422X-8-240

**Published:** 2011-05-19

**Authors:** Jiping Zhu, HongBo Zhou, Wei Zou, MeiLin Jin

**Affiliations:** 1 State Key Laboratory of Agricultural Microbiology, College of Veterinary Medicine, Huazhong Agricultural University, China

## Abstract

**Background:**

RAS, coded by *ras *proto-oncogenes, played an important role in signal transmission to regulate cell growth and differentiation. Host activation of RAS was significant for IFN-sensitive vaccinia virus (delE3L) or attenuate influenza virus in unallowable cells.

**Results:**

Huamn *NRAS *gene was activated by mutating in codon 61. Then the activation of NRAS was detected by western blot in MDCK cells. The delNS1 H5N1 influenza virus with deletion of NS1 eIF4GI binding domain was weak multiplication in MDCK cells. And the replication of delNS1 virus and expression of IFN-beta and IRF-3 were detected by Real-time PCR in MDCK cells infected with delNS1 virus. It was found that the delNS1 virus had a significant increase in MDCK cells when the NRAS was activated, and yet, expression of IRF-3 and IFN-beta were restrained.

**Conclusions:**

The study demonstrated that activated NRAS played an important part for delNS1 virus replication in MDCK cells. Activated NRAS might be down-regulating the expression of antiviral cellular factors in delNS1 virus infected cells.

## Background

The RAS family (NRAS, KRAS and HRAS) were members of the superfamily of small GTP/GDP-binding protein. They were known for their diverse intracellular signaling function in various organisms, such as controlling of cell proliferation, growth and apoptosis. Activating mutation in RAS occurred in up to 30% of human cancers. There were three ways to activate RAS protein: point mutation in *ras *gene, great amount expression of RAS and gene insertion or dislocation [1-3]. It was often found point mutations of codons 12, 13 or 61 in *ras *gene [4,5]. Constitutively active RAS, like the V12 mutation and V61 mutation, was predominantly binding with GTP, because of lower intrinsic GTPase activity and its resistance to the GTPase-stimulating activity of RASGAPs [6,7]. Activated RAS displayed oncogenic potential, being assistances of several downstream effectors including Raf, phosphatidylinositol 3-kinase and etc [8,9]. In RAS-transformed cells, activated RAS protein rendered cells permissive to defected virus, like delNS1 influenza virus (an influenza virus lacking the NS1 open reading frame), by a mechanism that involved induction of cellular inhibitor of PKR [10].

The nonstructural protein (NS1) of influenza virus was an important regulatory protein in virus replication and multiplication especially preventing host antivirus action. Previous studies suggested that binding of dsRNA by the NS1 protein blocked the action of the interferon (IFN)-inducible dsRNA-dependent protein kinase (PKR) [11,12]. The eIF4GI binding domain-truncated influenza virus did not efficiently replicate and was impaired in its ability to inhibit IFN production in vitro [13].

Previously, the delNS1 influenza virus (an influenza virus lacking the NS1 open reading frame) selectively replicated in the NRAS-expressing melanomas and had tumor-ablative potentials [10]. They reasoned that an activated ras-signaling pathway and inactivated PKR might allow replication of the delNS1 virus. In present study, NRAS was activated through point mutation in codon 61 (named NRAS (K61)). The replication of delNS1 virus was higher lever in MDCK transfected NRAS (K61). But the expression of IFN was inhibited in cells which had activated NRAS and virus incubating. Therefore, NRAS (K61) transient expressing in MDCK cells might make up for the deficiency of delNS1 influenza virus through inhibiting IFN effect.

## Methods

### Cell culture and Virus

A549 cells were maintained in F12 medium (SIGMA) supplemented with 10% FBS. And Madin-Darby canine kidney (MDCK) cells were in Dulbecco's modified Eagle's medium (DMEM) (Invitrogen) with 10% FBS. DelNS1 influenza virus (H5N1) with the deletion of NS1 eIF4GI binding domain was described previously [13].

### Construction of mutated plasmids

The *NRAS *ORF gene was amplified from A549 cells. And then the *NRAS *gene mutated in codons 61 (Q-K) by point mutation. PcDNA3.1 (+)-NRAS (K61) was made by inserting the NRAS.

### Plasmid transfections and virus infection

Liposomes (Invitrogen) were mixed with 3 μg DNA. And then, the transfection of MDCK cells was followed the manufacturer's instruction. After 12 h of transfection, MDCK in 6-well plates were incubated with delNS1 virus at a multiplicity of infection (MOI) of 0.1 in DMEM with FBS free at 37°C. One hour later, virus-containing solutions were removed by washing the cells twice with PBS. Infected cells were incubated in maintain medium with 2% FBS in a CO2 incubator at 37°C. Cell Supernatants and cells were harvested at 12 h, 24 h and 36 h after infection for western blot analysis and real time PCR. Experiments with delNS1 H5N1 virus were conducted in biosafety level 3+ containment facility.

### Apoptosis detection

After 36 h of transfected with NRAS (K61), cells' apoptosis was detected by cell death detection kit (Roche) following the manufacturer's instruction.

### Western blot analysis

Cells were harvested at 36 hours after transfection of NRAS (K61). Whole cells were lysed using RIPA buffer containing the cocktail of protease inhibitors (Roche Molecular Biology). Total protein was loaded on to SDS-PAGE and transferred on to nitrocellulose paper. The blot was incubated with the NRAS-specific monoclonal antibody F155 (Santa Cruz Biotechnology) followed by matching horseradish peroxidase-conjugated secondary antibody. Protein bands were visualized by enhanced chemiluminescence (Thermo).

### Real-time quantitative PCR for IFN-beta, IRF-3 and delNS1 virus production

To quantify the expressing levels of IFN-beta, IRF-3 and the replication of delNS1 influenza virus produced in NRAS (K61) activated cells infected with the delNS1 influenza viruses, a real-time quantitative PCR assay was used for IFN-beta, IRF-3 and NP. Total RNA was extracted from the MDCK cells with the RNA isolation kit (Qiagen), following the manufacturer's instruction. Total RNA (1 μg) was used for the synthesis of the first strand of cDNA. Reverse transcription (RT) was then carried out using the AMV Reverse Transcriptase XL kit (Takara). Real-time quantitative polymerase chain reaction (PCR) was conducted with 1 μl cDNA in a total volume of 25 μl with the iQ SYBR Green supermix (Bio-Rad), following the manufacturer's instruction. Relative expression values were normalized using a beta-actin internal control. The following oligonucleotide primer pairs were examined: beta-actin forward CGAGACATTCAACACCCCAAC, beta-actin reverse AGCCAGGTCCAGACGCAAG, IFN-beta forward CGCCGCAAGGACAGGATA, IFN-beta reverse ACGGGAGGTTCA-CAAGAAGGTT, IRF-3 forward GTTGGTAACAGACAAGACCGTGAT, IRF-3 reverse GAGTGTCGGCTTCCTTTGATG, NP forward TATTCGTCTCAGGGAGC-AAAAGCAGGGTA, and NP reverse ATATCGTCTCGTATTAGTAGAAACAAG-GGTATTTTT.

## Results

### Overexpression of mutated NRAS (K61) in MDCK cells

**Figure 1:**
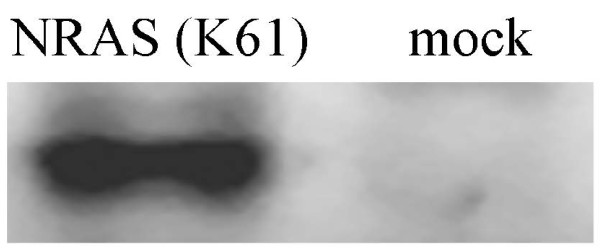
**Expression of NRAS (K61) in MDCK cells**. MDCK cells were transfected with 3 μg of pCDNA3.1 (+)-NRAS (K61). At 36 h posttransfection, cells were collected and lysed in SDS-PAGE gel loading buffer. Protein was detected by Western blotting by using N-ras-specific monoclonal antibody F155. A clear band could be seen in the blot.

**Figure 2:**
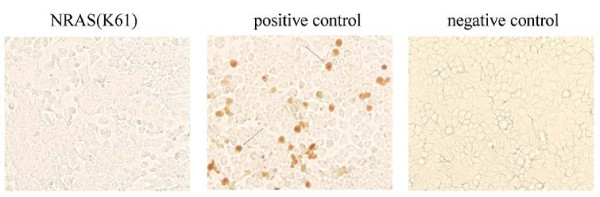
**Cells transfected with NRAS (K61) were not resulted in apoptosis**. After 36 h of transfection with NRAS (K61), MDCK was changed into comet cells. Cell death detection kit for immunohistochemical detection and quantification of apoptosis at single cell level, based on labeling of DNA strand breaks (TUNEL technology): analysis by light microscopy. In positive control, cells were incubated with DNase I recombinant for 10 min to induce DNA strand breaks. Arrow tip marked the apoptosis cells. For the NRAS (K61) transfected cells, there were no bright brown particles.

In order to obtain the activated human NRAS protein, the *NRAS *gene open reading frame was amplified from A549 cells and mutated on the 61 codon. The overexpression of the NRAS (K61) was examined in MDCK cells. The blot was shown that lysine mutation in condon 61 activated NRAS (Figure 1). Interestingly, it was observed great changing of cell morphology and disrupting of cell-cell adhesion. And apoptosis was not dectected in NRAS (K61) transfected cells (Figure 2).

### IFN-beta and IRF-3 gene expression and delNS1 virus multiplication in MDCK cells

**Figure 3:**
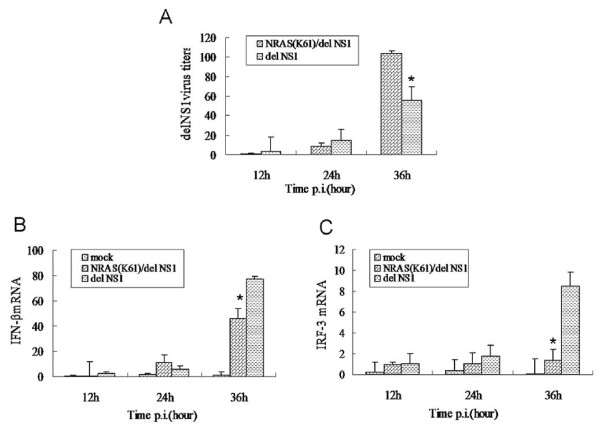
**NRAS (K61) transfected cells showed increases of delNS1 virus (A) and lower activation of IFN-beta(B) and IRF-3 (C)**. (A, B and C) At the times after infection indicated, cells were collected, and the total RNA was extracted. The amount of NP, IFN-beta and IRF-3 mRNA was determined by using quantitative RT-PCR. *Significant differences (p < 0:05, Manne Whitney test).

MDCK cells were infected by delNS1 virus after 12 h of transfection with NRAS (K61). Supernatants and cells were collected at different time points postinfection. The titers of delNS1 virus were detected by real-time PCR in MDCK cells transfected with NRAS (K61). These analyses showed that delNS1 virus had a great increase in transfected NRAS (K61) cells than not at 36 h p.i (p < 0.05). But the replication of the delNS1 virus was not obvious difference at 12 h p.i. and 24 h p.i. whether transfected NRAS (K61) or not (Figure 3A). IFN-beta and IRF-3 was also determined by the same way. At 12 h or 24 h p.i., there was no significant difference in the levels of IFN-beta and IRF-3 between transfected NRAS (K61) cells and the mock. The expression of IFN-beta and IRF-3, compared with the un-transfected MDCK cells, was repressed at 36 h postinfection of the delNS1 virus for the cells transfected NRAS (K61) (p < 0.05) (Figure 3B and 3C).

## Discussion

RAS was a member of a small GTPase superfamily, which could regulate a series of cell proliferation and apoptosis. In the present study, it was found that transient expression of oncogenic NRAS (K61) in MDCK cells influenced cell morphology and cell-cell contacts, leading to the production of comet cell. However, the typical apoptosis of the cells was not observed. It might be the way that constitutive activation of RAS resisted cell death with the change of cell morphology. In mouse cells, a transient increase in the cytoplasmic level of oncogenic H-Ras (H-Ras (V12)) led to disintegrate of E-cadherin complexes and activated of beta-catenin-dependent transcription in gene reporter assays [14].

RAS was activated in host, favorably for variety viral infection. In the HIV-1 model, downstream signaling pathways of RAS activation was beneficial to viral replication [15]. Similarly, some other viruses also interacted with RAS, such as influenza virus (delNS1 strain), hepatitis B virus, reovirus, vesicular stomatitis virus (VSV) and adenovirus (VAI mutant) [10,16-19]. Activated RAS was participated in different signaling pathway and exercised multiple functions in various virus proliferations. In the activated form of RAS, it was shown an increase of the HBV X protein. In addition, activation of the RAS signal transduction pathway could enhance the central transcription factor, TBP, and the induce of RNA pol III-dependent gene activity in Hepatitis B Virus infected cells, which increased the viral replication [16]. Cells with an activated RAS/RAF/MEK signaling cascade allowed propagation of viruses in the presence of IFN. Vesicular stomatitis virus (VSV) or an IFN-sensitive vaccinia virus (delE3L) infected RAS-transformed (RasV12) cells in the presence of alpha interferon, both of which were found to similarly flourish in cells regardless of the presence of IFN [20]. In this study, it was observed prominent increase of delNS1 virus in the cell of activated NRAS (K61), which meant effective replication of delNS1 virus.

In the present study, the delNS1 H5N1 influenza viruses with the deletion of eIF4GI binding domain displayed attenuated virulence in MDCK cells. In addition, this delNS1 H5N1 strain could stimulate strong response of IFN. DelNS1 virus lacking eIF4GI binding domain was weak multiplication in MDCK cells because of omnipresent dsRNA-dependent protein kinase (PKR) inducing antivirus action or unable preferential translation viral mRNA [21,22]. Recent studies were shown that the NS1 protein of influenza A virus was a major force to antagonize activation of PKR and the expression of early defense genes, including IFN-beta [11,12,23,24]. IRF-3, as a transcription factor required for the induction of IFN-beta, also could be inhibited by NS1 protein [23,25-28].

In current study, growth of delNS1 virus was dependent on expression of NRAS (K61) in MDCK cells. And it was shown that activated RAS down-regulated IFN-induced antiviral responses. It was demonstrated that activated RAS had been entangled in the negative regulation of the IFN response. Previous study showed that the active K-RAS restrained the IFN-gamma-activated sequence-mediated transcription of IFN-gamma in human cancer cells [29]. And viral oncogene (v-RAS) was transfected in BALB/c-3T3 cells leading to inhibition of induction of MHC I by IFN-alpha [30]. Some other studies also reported that the suppression of the IFN response by activated RAS could be a common mechanism which was exploited by some oncolytic viruses. This effect was mediated at least in part by the ability of the RAS/ERK signaling pathway to inhibit activation of IRF-3 [18]. IRF-1, initially described as a transactivator of the IFN-beta gene, was deregulated in RAS-transformed mouse fibroblasts (RS485) [31-33]. The cellular activity of the RAS/MEK pathway might influence cellular susceptibility to IFN and be the making of host susceptibility to virus [34].

## Conclusions

In conclusion, this study demonstrated that activated RAS could help defective influenza virus propagation. It is speculated that constitutive activation of RAS inhibited IFN antivirus defenses and afforded more favorable environment for replication of delNS1 influenza virus. The virus-infected RAS activated cells were affected by the IFN in a complicated way, suggesting that RAS played a significant role in virus infection, preserving the cells, cell transformation and so on. The mode of action of RAS needs further research.

## Competing interests

The authors declare that they have no competing interests.

## Authors' contributions

JZ and WZ did the data base analyses. JZ drafted and finalized the manuscript. JZ and HZ carried out the construction of plasmid, influenza virus infection and plasmid transform. JZ and WZ performed the western blot and real-time PCR. HZ and MJ designed the study and supervised the experiments. All authors read and approved the manuscript.
